# Perinatal outcomes of maternal overweight and obesity in term infants: a population-based cohort study in Canada

**DOI:** 10.1038/srep09334

**Published:** 2015-03-20

**Authors:** Angela Elena Vinturache, Sheila McDonald, Donna Slater, Suzanne Tough

**Affiliations:** 1 Department of Paediatrics, Cumming School of Medicine, University of Calgary, Calgary, Alberta, T2N 4N1, Canada; 2 Department of Physiology & Pharmacology, Cumming School of Medicine, University of Calgary, Calgary, Alberta, T2N 4N1, Canada; 3 Alberta Children's Hospital Research Institute for Child and Maternal Health (ACHRI), Cumming School of Medicine, University of Calgary, Calgary, Alberta, T2N 4N1, Canada

## Abstract

The objective of this study was to assess the impact of increased pre-pregnancy maternal body mass index (BMI) on perinatal outcomes in term, singleton pregnancies who received prenatal care in community-based practices. The sample of 1996 infants included in the study was drawn from the All Our Babies Study, a prospective pregnancy cohort from Calgary. Multivariable logistic regression explored the relationship between the main outcomes, infant birth weight, Apgar score, admission to neonatal intensive care (NICU) and newborn duration of hospitalization, and BMI prior to pregnancy. Approximately 10% of the infants were macrosoms, 1.5% had a low Apgar score (<7 at 5 min), 6% were admitted to intensive care and 96% were discharged within 48 h after delivery. Although the infants of overweight and obese women were more likely to have increased birth weight as compared to infants of normal weight women, there were no differences in Apgar score, admission to NICU, or length of postnatal hospital stay among groups. This study suggests that in otherwise healthy term, singleton pregnancies, obesity does not seem to increase the risk of severe fetal impairment, neonatal admission to intensive care or duration of postnatal hospitalization.

Obesity has become a worldwide epidemic and an important health concern due to increased risk of serious health consequences that encompass metabolic and cardiovascular complications^1^,^2^. Consistent with the trend observed in the general population, the rates of overweight and obesity in women of childbearing age are increasing rapidly. In Canada, over 10% of women of reproductive age are obese^2^,^3^. In USA, more than half of pregnant women are obese or overweight, and around 8% are morbidly obese^4^. Maternal overweight and obesity increase the risks of complications during pregnancy and delivery^5^,^6^,^7^,^8^ as well as neonatal and infant morbidity and mortality^9^,^10^,^11^,^12^. Obesity and morbid obesity in pregnancy has been linked to antepartum stillbirth, large-for-gestational-age (LGA), shoulder dystocia, meconium aspiration, fetal distress^13^ and 5 min Apgar score <4^14^. Other perinatal problems associated with maternal obesity include congenital anomalies^14^,^15^, birth trauma, birth asphyxia, and neonatal hypoglycemia^16^,^17^ although the underlying mechanisms of these associations are still uncertain^10^.

While evidence suggests that maternal obesity is a risk factor for adverse labour and delivery outcomes^18^,^19^,^20^ and increased health care service utilization at birth, it is not clear to what extent maternal BMI prior to pregnancy influence neonatal health. Several studies report on adverse perinatal outcomes in severely obese mothers^17^,^21^ but few describe the impact of maternal obesity from the remainder of BMI classification categories on Apgar score, newborn admission to the intensive care or neonatal length of stay^16^. Furthermore, although fetal macrosomia (birth weight over 4,000 g) is recognized as a clinical problem in obstetrics, there are no specific recommendations for intrapartum care of these infants. In addition, most studies describe the perinatal outcomes in heterogeneous populations, including both preterm and term pregnancies^21^; however, the overall morbidity at birth may be different in obese women from otherwise healthy pregnancy as compared to women with additional risk factors. Taken together, this suggests that different populations, different classifications of obesity and probably other confounding factors may impact on the commonly perceived high risk of adverse perinatal outcomes in obese women.

Therefore, this study aimed to determine the impact of maternal pre-pregnancy BMI on perinatal outcomes in a population of term, singleton, cephalic pregnancies who received prenatal care in community health care centers and obstetrical care in labour and at delivery in tertiary centers. The primary question to be answered was to determine the relationships between increased maternal body weight before pregnancy and fetal vitality and wellbeing at delivery defined as Apgar score, neonatal intensive care admission and length of hospital stay. This study also develops previous work regarding the influence of maternal BMI on fetal intrauterine growth.

## Methods

The data for this study was drawn from the All Our Babies Study (AOB), a prospective community-based pregnancy cohort of approximately 3388 women based in Calgary, Alberta, Canada^40^. Women were enrolled in the AOB study from December 2008 to July 2010, at less than 24 weeks of gestation and completed three questionnaires at 24 and 32 weeks of gestation, and 4 months post-partum. Self-reported data on demographics, lifestyle, health care utilization, pregnancy history, and physical and mental health were collected and this information was linked to the obstetric electronic medical records from labour and delivery. Maternal pre-pregnancy body mass index (BMI) was determined from the maternal self-reported height and pre-pregnancy weight and was calculated as weight prior to pregnancy (kg) divided by height (m) squared. The subjects were divided into groups according to pre-pregnancy BMI using body mass categories as defined by World Health Organization^41^ and Health Canada Guidelines recommendations^42^: underweight (BMI < 18.50 kg/m^2^), normal weight (BMI 18.50–24.99 kg/m^2^), overweight (BMI 25.00–29.99 kg/m^2^), and obese (BMI ≥ 30.00 kg/m^2^).

Information about socio-demographic characteristics and length of hospital stay were obtained from self-reported questionnaires. The socio-demographic characteristics included maternal age (less than 34 and more than 35 years of age), marital status (single, married, or common-law), level of education (high school or less, some or completed post-secondary), annual household income (less than $40,000, $40,000–79,999, and above $80, 000), and ethnicity (Caucasian, non-Caucasian). Information on the obstetrical characteristics and labour and delivery outcomes extracted from medical records included: obstetrical history (parity, history of large-for-gestational-age infants), pregnancy complications (pregnancy-induced hypertension, preeclampsia, gestational diabetes mellitus), type of labour (induced or spontaneous), mode of delivery (spontaneous vaginal delivery, emergency caesarean section, assisted vaginal delivery (forceps or vacuum), gestational age (GA) at delivery (in weeks), newborn gender and birth weight (in grams), Apgar score at 5 min, resuscitation at birth, presence of meconium in amniotic fluid, admission to NICU, and newborn length of hospital stay (LOS). For neonatal birth weight classification we used the 10th and the 90th birth weight percentile as cut-offs for defining small for gestational age (SGA) and large for gestational age (LGA), respectively.

For this study, only women who had singletons, cephalic presentations, delivered at term (≥37 weeks gestation), had a pre-pregnancy BMI ≥ 18.5 kg/m^2^, and their survey data could be linked to the medical records at labour and delivery were included in this analysis (N = 1996).

The main independent variable was maternal pre-pregnancy BMI, classified as normal weight, overweight, and obese, with normal weight as reference category. The main outcomes of interest were infant birth weight (stratified as ≥4000 g, macrosomia; and <4000 g)^43^, Apgar score at 5 min (<7, low score; ≥7)^44^, admission to NICU (admitted; not admitted) and newborn duration of hospitalization (≤/>24 h; ≤/>48 h)^36^.

### Ethics

All women who participated in this study received prenatal care in community health care settings and delivered in urban tertiary care centers affiliated to University of Calgary. The Conjoint Health Research Ethics Board of the University of Calgary provided ethical approval for this study. The methods of this study were carried out in accordance with the approved guidelines. Written informed consent was obtained from the study participants at the time of recruitment, who were provided copies of the consent forms for their records.

### Statistical analysis

Descriptive statistics were produced for all study variables. Medians and interquartile ranges (IQR) were used to summarize the continuous data, and frequency distributions were used to summarize the categorical data. Chi-square test was used to examine the associations between different BMI groups and the socio-demographic characteristics of the study population. Bivariate regression analyses assessed the likelihood of obstetrical and neonatal complications in women with increased pre-pregnancy BMI as compared to women with normal weight. Multivariable logistic regression analyses were performed to determine the relationship between perinatal outcomes (i.e. fetal macrosomia, Apgar score at 5 min, newborn admission to NICU, and infant length of postnatal hospitalization) and maternal pre-pregnancy BMI categories. A hierarchical building model strategy was used and blocks of variables were entered in the following order: demographic variables, obstetrical variables, and neonatal variables. Maternal BMI variables were entered at the first step in all models regardless of their bivariate statistical significance. Odds ratios and 95% confidence intervals were presented for final models; a value of p < 0.05 was considered statistically significant. All statistical analyses were performed using the SPSS for Windows package, versions 20 (IBM SPSS, Chicago, IL).

## Results

### Population demographic and obstetrical characteristics

Table 1 summarizes the demographics of the study population. Of the 1996 participants included in the study, 1313 (65.8%) were normal weight, 427 (23.6%) were overweight and 211 (10.6%) were obese (the anthropometric measures presented in Table 1), from which 31 had BMI ≥ 40 kg/m^2^. The majority of women in the study were Caucasian (1602; 80.4%), younger than 35 years (1563, 78.3%), in a marital or common-law relationship (1901; 95.4%), had a household income higher than $ 80,000 (1406, 72.5%), and level of education higher than high school (1802, 90.5%). No differences were observed concerning maternal age at delivery, marital status and household income between the BMI categories, although obese women were more likely to be Caucasian and have attained lower levels of education than women with normal weight prior to pregnancy.

Comparison of obstetrical characteristics and maternal outcomes in labour and at delivery based on pre-pregnancy BMI are presented in Table 2. There was no difference in parity between the three groups of women. However, the history of delivery of a LGA baby was more frequent in obese and overweight women than in women of normal weight. A graded association was seen for the likelihood of pregnancy complications including pregnancy-induced hypertension, preeclampsia, eclampsia, diabetes mellitus, and placenta praevia according to BMI category. The likelihood of spontaneous onset of labour decreased with increasing BMI, with obese women having the highest risk for labour induction (OR 2.5, 95% CI 1.8–3.3); almost half of the obese women had their labour induced. In addition, these women were more likely to deliver by emergency caesarean section (OR 2.5, 95% CI 1.6–3.8) and less likely to deliver by forceps or vacuum (OR 0.4, 95% CI 0.2–0.8) compared to normal weight women.

### Newborn characteristics

Newborn characteristics are illustrated in Table 2. Fifty two percent of newborns from the cohort were male. No difference was observed in distribution of newborn gender among the BMI categories. Whereas 62.5% of the babies born to normal weight mothers were delivered full term (39^0/7^– 40^6/7^ weeks gestation), the fetuses from overweight pregnancies were more likely to be delivered late term (41^0/7^– 41^6/^weeks) (OR 1.5, CI 1.1–1.9) and the fetuses from obese pregnancies early term (37^0/7^– 38^6/7^ weeks) (OR 1.9, CI 1.4–2.6). The median weight at birth was 3395 g (IQR 591), with newborns of overweight and obese women being heavier than newborns of normal weight women (3473 g (IQR 643) and, respectively, 3476 g (IQR 686) (p < 0.05 for both). Although no differences were observed between groups concerning the lower end of the birth weight curve (LBW and SGA), a linear relationship was observed between fetal macrosomia and maternal BMI. Infants of obese mothers were more likely to undergo resuscitation at birth (OR 1.4, CI 1.0–1.9).

### Perinatal outcomes

Table 3 presents the adjusted models of the associations between perinatal outcomes including macrosomia, Apgar score, NICU admission and postnatal hospital stay and maternal BMI prior to pregnancy; the variables included in the models are presented in the supplemental data, Table S1. In multivariable regression analysis, the adjusted odds for delivering a macrosomic infant increased by half in overweight (OR 1.4, CI 1.1–2.1) and by two fold in obese women (OR 2.0, CI 1.2–3.1) as compared to lean women. Caucasian ethnicity, multiparity, and gestational age at delivery of 41 weeks or more were independently associated with the risk of macrosomia. In our model, history of delivery of a LGA infant was the strongest predictor of a macrosomic infant, increasing the risk about five times (OR = 4.9, CI 1.8–13.3) (Supplemental data, Table S1).

In our cohort of singleton pregnancies delivered at term in cephalic presentation, no differences were observed in the Apgar score, NICU admission and 48 h length of postnatal hospital stay between the infants of overweight and obese women as compared to normal weight women. Although we did not observe associations between the aforementioned perinatal outcomes and pre-pregnancy BMI (Table 3), we performed multivariable analyses to control for any possible confounding effect and identify obstetrical factors that may influence these perinatal outcomes. Pregnancy complications (included in the model as composite variable of pregnancy-induced hypertension, preeclampsia, and gestational diabetes), delivery by emergency caesarean, and presence of meconium in amniotic fluid were predictors of low Apgar score, each increasing the risk by almost three folds (Supplemental data, Table S1). The median Apgar score at 5 min of the cohort was 9 (IQR 0) with only 1.5% of the neonates scoring lower than 7. The lowest Apgar score recorded was 2. The majority (80%) of the newborns with low Apgar were born to mothers with normal weight. Almost 6% (118) of the newborns were admitted to specialized care in the intensive care ward. Maternal age younger than 35 years, nulliparity, delivery by emergency caesarean, Apgar score <7 at 5 min, resuscitation at birth and presence of congenital anomalies increased the odds of admission to NICU (Supplemental data, Table S1). Low Apgar score was the strongest predictor of NICU admission, increasing the risk almost 15 times (adjusted OR 14.9, CI 6.6–33.4).

Fifty five percent of the infants from our population were discharged from the tertiary maternity hospitals within 24 h of delivery, and 96% of infants were discharged within 48 h. All 13 infants in need of further acute medical care were transferred to specialized hospital services within 48 h of delivery. Maternal overweight was independently associated with early hospital discharge (Table 3), within 24 h of delivery. Parity and spontaneous vaginal delivery were the strongest predictors of short hospital stay (less than 48 h) (Supplemental data, Table S1), whereas maternal obesity, in spite of increasing the risks of pregnancy complications and adverse birth outcomes, was not associated with newborn length of stay.

## Discussion

This prospective community-based study suggests that being overweight or obese does not independently increase the risks of low Apgar score at birth, admission to neonatal intensive care or increased postnatal hospital stay. However, our study demonstrates that increased maternal BMI before conception influences intrauterine growth and infant weight at delivery and labour and delivery outcomes. Importantly, the linear association between BMI and the risk of pregnancy complications, active management in labour and at delivery, including labour induction and surgical delivery, are evidence that contribute to the body of knowledge of the adverse effects of obesity on pregnancy and maternal health.

As expected, using birth weight as a proxy for intrauterine growth, we found that the risk of delivery of an infant with birth weight >4000 g increases with increasing maternal BMI prior to conception. This data aligns with other research that suggests a 1.5 to 2.3 increase in the adjusted odds of delivering large for date infants in obese women^17^,^22^,^23^. Maternal obesity is a well-known risk factor for accelerated intrauterine growth, fetal macrosomia being consistently reported to associate with increased maternal body weight^17^,^22^,^23^.

In this study, we used Apgar score, a conventionally, standardised tool that evaluates physical condition at birth as an indicator of the impact of maternal BMI on the vitality and wellbeing of the newborn. Low Apgar scores are predictive of adverse immediate (i.e. NICU admission, increased hospitalization) and long term outcomes (cognitive impairment^24^, neurologic sequeale^25^). No association was evident in our study between maternal overweight and obesity and low Apgar score at 5 min. Likewise, two other large studies evaluating fetal wellbeing in healthy obese women^16^,^26^, found no differences in neonatal Apgar scores between lean and obese women. In contrast, studies in large cohorts from British and Danish populations reported increasing risk of low Apgar scores (<7) with increasing maternal BMI^20^,^27^, after controlling for pregnancy complications. In a recent Swedish study by Persson *et al*^10^ maternal overweight and obesity associated with increased risk of Apgar scores <3 at 5 and 10 minutes, after controlling for congenital malformations and pregnancy complications, obesity-related conditions known to increase the risk of fetal hypoxia^10^. In our analyses, both type of labour and mode of delivery emerged as strong predictors of low Apgar scores, despite availability of advanced obstetrical care in tertiary hospitals. The inconsistency in findings of the associations between maternal BMI and Apgar scores may result from the differences between the characteristics of the populations or infants studied, Apgar score assessement, or BMI classification categories. Of note, in several of the pregnancy cohorts mentioned above, the studies spanned several years^27^ or even decades^10^, possibly reflecting changing clinical practices over time. In contrast, our study covered a short, defined interval when major changes in obstetrical practice were less likely to occur. Furthermore, we have excluded all preterm births from our study because the infants from these pregnancies are known to be at increased risks of poor neonatal outcomes. In contrast with above-mentioned studies, we report low prevalence of Apgar <7 (1.6%) that is, however, representative for Calgary metropolitan area (1.7%) and the province of Alberta (2.6%), and this may limit generalization of our findings^28^. Additional studies are warranted to evaluate the impact of maternal obesity on Apgar score at delivery in specific populations.

In contrast with earlier studies that report an increased risk of admission to neonatal care for the children of obese mothers^16^,^29^,^30^, we found no difference in NICU admission rates of the infants of obese and overweight women in comparison with infants of normal weight women. Other recent studies report no differences in NICU admission rates of neonates of severe and morbidly obese or normal weight mothers, in agreement with our findings^17^. The discrepancy in the findings may be due to differences in the identification and prioritization of the infants who can benefit from ICU care. That is, the reasons for admision to NICU, illness or surveillance, abide different meanings concerning the health of the infant. For instance, in a large German cohort, Kalk *et al* conclude that the increased admission rates of newborns of obese mothers are determined by impaired glucose metabolism in these infants, thus requiring neonatal ward surveillance, and are not due to severe fetal impairment^30^. We were unable to asses the indications for neonatal ward admission in our study and, therefore, could not fully evaluate the reasons for the inconsistency between the findings. However, in our analysis low Apgar score emerged as an independent risk factor for admision to NICU. Several other studies report low Apgar scores, hence neonatal illness, as criteria for admission to care in NICU in overweight and obese mothers^16^,^29^,^30^. Most studies report the NICU admision rates in more heterogenous populations, including preterms, or clinical management in centers of various levels of neonatal care, which may limit the comparisons with our selected population who delivered at term, in tertiary centers with similar scope of practice. Such an assumption is supported by the percentages of neonatal admissions reported: In the study by Kalk *et al* about 25% of the children were admitted to NICU^30^, whereas Usha Kiran *et al* report only 2.5–3.8% admissions in a population selected on criteria similar with ours^16^.

Other studies have found an independent association between morbid obesity and LOS, or report that the increased hospital stay in overweight and obese pregnancies was largely mediated by pregnancy complications^29^,^31^,^32^. In our study, pregnancy complications did not increase the odds of duration of hospitalization among otherwise healthy women with higher BMI. Mamun *et al* concur, reporting on association between LOS and gestational weight gain, independent of pregnancy complications and mode of delivery^33^. Beside BMI classification, the variability of clinical practice regarding the appropriate duration of hospitalization at birth and the mode of LOS reporting (days vs hours) may explain, at least partially, the disagreement between findings from different studies including ours. The appropriate LOS has been a controversial issue for decades, a number of factors including maternal and neonatal health, health services and community resources playing a major role in the decision of maternal-child dyad hospital discharge. Currently, there is a trend of early hospital discharge for both mother and child following uncomplicated childbirth^34^,^35^. Among Canadian provinces and territories, in the period 2002–2003 to 2004–2005, the province of Alberta had the largest proportion (47.8 for 100 hospital live births) of term newborns discharged within two days of birth^36^.

A major strength of this study is represented by the prospective design of the study cohort. The relatively large sample size of the cohort permitted selection of a phenotyped population to study the effects of overweight and obesity on outcomes at birth. Similar to all observational studies, our study has inherent limitations such as potential for misclasification due to self-report; however, a number of our variable were extracted from the medical records which may decrease the risk for this bias^37^,^38^,^39^. Furthermore, due to the inclusion criteria, women with potential high risk of adverse perinatal outocmes (i.e. underweight at conception) were excluded from the study, which precluded examination of this weight category on outcomes. In addition, because of limited number of women with BMI > 40.0, we could not study separately the outcomes in all BMI obese categories. Therefore, our findings might not apply to morbidly obese women.

In conclusion, this study demonstrates that among otherwise healthy overweight and obese women who deliver at term, cephalic, singleton infants, pre-pregnancy BMI was not associated with low Apgar scores or increased health service utilization at birth. The relatively limited impact of obesity on perinatal outocomes found in the present study may suggest increased awareness of health care providers of the potential risks of maternal and perinatal morbidity in women with increased BMI. In addition, the growing literature on the subject over the past two decades and release of new and updated clinical guidelines and principle of practice may have contributed to better management and increased quality of care of healhty obese women who become pregnant. Moreover, our findings may suggest that perinatal care in tertiary centers may offset the risk factors of poor outcomes, as suggested previoulsy by Stepan *et al.*^21^. This is an important aspect of this research which may have implications with respect to the costs of perinatal health care and decision of postnatal hospital discharge in pregnant overweight and obese women who are otherwise healthy.

## Author Contributions

A.V. conceived and designed the study with input from S.T. and S.M.W. A.V. undertook the analysis and interpretation of the data with input from S.T., S.M.W. and D.S. A.V. wrote the first draft of the manuscript. S.C.T. was involved in AOB study design and acquisition of funding, and is responsible for overall integrity, progress and timely completion of the AOB study. S.W.M. was responsible for the management, coding, linkage of cohort data. D.S. is an investigator with AOB study. All authors participated in the editing of the manuscript and approved the final version for publication.

## Supplementary Material

Supplementary Information Supplementary data

## Figures and Tables

**Table 1:** Descriptive data of the study population across BMI categories§

	Pre-pregnancy BMI			
	Normal weight	Overweight	Obese	p-value
Percent of study sample n (%)	1313 (65.8)	472 (23.6)	211 (10.6)	
Maternal age at birth (years), median(IQR)	31.0 (6)	31.4 (5)	30.5 (7)	0.326
Maternal pre-pregnancy weight (kg), median(IQR)	60.0 (9.4)	73.3 (9.9)	93.1 (18.0)	**<0.001**
Maternal height (cm), median(IQR)	166.1 (8.1)	165.1 (10.1)	165.1 (10.1)	0.585
Ethnicity n (%)				
Caucasian	1036 (78.5)	394 (83.1)	186 (86.1)	**0.004**
Non-Caucasian	284 (21.5)	80 (16.9)	30 (13.9)	
Marital status n (%)				
Single	59 (4.5)	21 (4.4)	12 (5.6)	0.734
Married/common low	1251 (95.5)	451 (95.6)	199 (94.3)	
Income n (%)				
≤$39,000	87 (6.8)	34 (7.4)	18 (8.7)	0.125
$40,000–$79,999	243 (19.0)	98 (21.4)	53 (25.7)	
≥$80,000	946 (74.1)	325 (71.1)	135 (65.5)	
Education n (%)				
Less than high school or high school graduate	108 (8.3)	47 (10.0)	35 (16.6)	**0.001**
Some or completed post-secondary	1201 (91.7)	425 (90.0)	176 (83.4)	

^§^Normal weight = BMI 18.50–24.99 kg/m^2^; Overweight = BMI 25.00–29.99 kg/m^2^, Obese = BMI > 30.00 kg/m^2^.

**Table 2:** Comparison of obstetrical and neonatal characteristics between pre-pregnancy BMI categories§

	Normal weight	Overweight	OR (CI)	p-value	Obese	OR (CI)	p-value
Multiparity	614 (46.8)	231 (48.9)	1.1 (0.8–1.3)	0.416	106 (50.2)	1.1 (0.8–1.5)	0.523
History of LGA	7 (0.5)	8 (1.7)	**3.2 (1.1–8.9)**	0.025*	4 (1.9)	**3.6 (1.0–12.4)**	0.042*
Pregnancy complications^1^	173 (13.2)	106 (22.5)	**1.9 (1.4–2.4)**	<0.001*	66 (31.3)	**3.0 (2.1–4.2)**	<0.001*
Gestational age at delivery(weeks)							
Early term 37^0/7^– 38^6/7^	295 (22.5)	108 (22.9)	1.0 (0.7–1.3)	0.854	75 (35.5)	**1.9 (1.4–2.6)**	<0.001*
Full term 39^0/7^– 40^6/7^	820 (62.5)	266 (56.4)	**0.7 (0.6–0.9)**	0.020*	105 (49.8)	**0.6 (0.4–0.8)**	0.001*
Late term 41^0/7^– 41^6/7^	194 (14.8)	97 (20.6)	**1.5 (1.1–1.9)**	0.004*	31 (14.7)	0.9 (0.6–1.5)	0.975
Postterm ≥42^0/7^	4 (0.3)	1 (0.2)	0.7 (0.07–6.2)	0.745	0 (0.0)		
Induction of labour	366 (27.9)	180 (38.1)	**1.5 (1.2–1.9)**	<0.001*	103 (48.8)	**2.5 (1.8–3.3)**	<0.001*
Maternal indication	74 (20.9)	50 (28.4)			43 (42.2)		
PROM	129 (36.4)	53 (30.1)			25 (24.5)		
Postterm pregnancy	88 (24.9)	46 (26.1)			20 (19.6)		
Fetal indication	32 (9.0)	15 (8.5)			7 (6.9)		
Amniotic fluid disorders	17 (4.8)	8 (4.5)			4 (3.9)		
Other	14 (4.0)	4 (2.3)			3 (2.9)		
Spontaneous vaginal delivery	1031 (78.5)	354 (75.0)	0.8 (0.6–1.0)	0.116	156 (73.9)	0.7 (0.5–1.0)	0.137
Assisted vaginal delivery (forceps and/or vacuum)	132 (10.7)	55 (12.5)	1.2 (0.8–1.6)	0.303	8 (4.4)	**0.4 (0.2–0.8)**	0.011*
Emergency caesarean section	82 (6.2)	33 (7.0)	1.1 (0.7–1.7)	0.571	30 (14.2)	**2.5 (1.6–3.8)**	<0.001*
Fetal distress	36 (46.2)	15 (51.7)			10 (34.5)		
Abnormal labour	34 (43.6)	14 (48.3)			16 (55.2)		
Maternal indication	7 (9.0)	0 (0.0)			3 (10.3)		
Other	1 (1.3)	0 (0.0)			0 (0.0)		
Newborn gender							
female	620 (47.2)	232 (49.2)	0.9 (07–1.1)	0.471	106 (50.2)	0.8 (0.6–1.1)	0.415
male	692 (52.8)	240 (50.8)			105 (49.8)		
SGA^2^	148 (12.0)	45 (10.2)	0.8 (0.5–1.1)	0.318	15 (7.5)	0.6 (0.3–1.0)	0.070
LBW^3^	23 (1.8)	10 (2.1)	1.2 (0.5–2.5)	0.612	4 (1.9)	1.0 (0.3–3.1)	0.883
LGA^4^	77 (6.2)	43 (9.8)	**1.6 (1.1–2.4)**	0.014*	25 (12.6)	**2.1 (1.3–3.4)**	0.001*
Meconium in amniotic fluid	250 (19.0)	112 (23.7)	**1.3 (1.0–1.7)**	0.030*	37 (17.5)	0.9 (0.6–1.3)	0.604
Resuscitation^5^	582 (44.3)	218 (46.2)	1.07 (0.8–1.3)	0.486	113 (53.6)	**1.4 (1.0–1.9)**	0.013*

Abbreviations: BMI, body mass index; OR(95%CI), unadjusted odds ratio (95% Confidence Interval);

^1^Pregnancy complications include: pregnancy-induced hypertension, preeclampsia, eclampsia, diabetes mellitus and placenta praevia;

^2^SGA, Small for Gestational Age, birth weight below 10th percentile of sex-specific birth weight;

^3^LBW, birth weight below 2500 g;

^4^LGA, Large for Gestational Age, birth weight above 90th percentile of sex-specific birth weight;

^5^Newborn resuscitation at birth included any of the following methods alone or combined: suction, oxygen, bag/mask, endotracheal tube, cardiopulmonary resuscitation, medication;

^§^Normal weight = BMI 18.50–24.99 kg/m^2^; Overweight = BMI 25.00–29.99 kg/m^2^, Obese = BMI > 30.00 kg/m^2^;

*p < 0.05 as compared to normal pre-pregnancy weight group (reference category).

**Table 3:** Perinatal outcomes across maternal pre-pregnancy BMI categories§

	Normal weight	Overweight‡			Obese‡		
	n (%)	n (%)	Crude OR (95%CI)	Adjusted OR (95%CI)	n (%)	Crude OR (95%CI)	Adjusted OR (95%CI)
Fetal macrosomia^1^	107 (8.1)	60 (12.7)	**1.6 (1.1–2.3)**	**1.4 (1.1–2.1)**	31 (14.7)	**1.9 (1.2–2.9)**	**2.0 (1.3–3.2)**
Apgar score <7 at 5 min^2^	23 (1.8)	4 (0.8)	2.0 (0.7–6.0)	2.0 (0.6–6.2)	2 (0.9)	1.8 (0.4–7.9)	1.9 (0.4–8.9)
NICU admission^3^	74 (5.6)	28 (5.9)	1.1 (0.6–1.6)	1.1 (0.7–1.8)	16 (7.6)	1.3 (0.7–2.4)	1.4 (0.7–2.6)
LOS^4^							
LOS ≤ 24 hs	351 (52.4)	135 (62.5)	**1.5 (1.1**–**2.1)**	**1.4 (1.1–2.1)**	48 (55.8)	1.1 (0.7–1.8)	1.0 (0.6–1.6)
LOS ≤ 48 hs	642 (95.8)	210 (97.2)	1.5 (0.6–3.7)	1.1 (0.4–2.8)	85 (98.8)	3.7 (0.5–27.6)	5.5 (0.4–61.7)

Abbreviations: BMI, body mass index; LOS, length of hospital stay; NICU, neonatal intensive care unit; OR, odds ratio; CI, confidence intervals.

^1^Fetal macrosomia defined as birth weight ≥4000 g; adjusted for maternal age, ethnicity, history of LGA, gestational age at delivery;

^2^Apgar score at 5 min less than 7; adjusted for pregnancy complications, type of labour onset and mode of delivery, presence of meconium in the amniotic fluid;

^3^Neonatal intensive care admission; adjusted for maternal age, parity, mode of delivery, Apgar score at 5 min, resuscitation at birth, congenital anomalies;

^4^Postnatal length of hospital stay; adjusted for maternal age, parity, mode of delivery, Apgar score at 5 min, and admission to NICU;

^‡^Reference category normal weight, BMI = 18.50–24.99 kg/m^2^ pre-pregnancy; ^§^Overweight = BMI 25.00–29.99 kg/m^2^, Obese = BMI > 30.00 kg/m^2^.
