# Supplementary material for: Perinatal outcomes of maternal overweight and obesity in term infants: a population-based cohort study in Canada
Source: Sci Rep. 2015 Mar 20;5:9334. doi: 10.1038/srep09334 (PMC4366803; doi:10.1038/srep09334)
Supplement: Supplementary Information — Supplementary data [file srep09334-s1.pdf]

Supplementary file

Title, Perinatal outcomes of maternal overweight and obesity in term infants: a population-based cohort study in Canada

Running title, Maternal BMI and perinatal outcomes

Authors, Angela Elena Vinturache[1,2,3], Sheila McDonald[1,3], Donna Slater[2,3], Suzanne Tough[1,3]

Affiliations:

[1]Department of Paediatrics

[2]Department of Physiology & Pharmacology

[3]Alberta Children's Hospital Research Institute for Child and Maternal Health (ACHRI)

Cumming School of Medicine, University of Calgary, Calgary, Alberta, T2N 4N1, Canada

Corresponding author: Angela Vinturache
Department of Paediatrics/Physiology&Pharmacology
Faculty of Medicine
University of Calgary
Child Development Centre
c/o 2888 Shaganappi Trail NW
Calgary, AB, T3B 6A8
Telephone: +1 403 955 5975
Email: aevintur@ucalgary.ca

Table S1. Risk factors for perinatal outcomes (macrosomia, low Apgar score, NICU admission, length of postnatal hospital stay) in term, singleton, low-risk pregnancies

Risk Factors	aOR	95%CI
**Macrosomia**		
Maternal overweight[1]	**1.5**	**1.1 - 2.1**
Maternal obesity[2]	**2.1**	**1.3 - 3.2**
Ethnicity[3]	**2.1**	**1.3 - 3.5**
Parity[4]	**1.8**	**1.3 - 2.5**
History of LGA	**4.9**	**1.8 - 13.3**
Gestational age[5]	**2.1**	**1.5 - 3.0**
**Apgar score < 7 at 5min**		
Pregnancy complications[6]	**3.0**	**1.0 - 8.3**
Labour induction	**0.3**	**0.09 - 0.9**
Emergency caesarean section	**3.1**	**1.3 - 7.4**
Meconium in amniotic fluid	**2.7**	**1.1 - 6.4**
**NICU admission**		
Maternal age[7]	**2.0**	**1.3 - 3.1**
Parity[8]	**1.7**	**1.0 - 2.6**
Emergency caesarean section	**2.8**	**1.5 - 3.5**
Low APGAR score at 5min[9]	**14.9**	**6.6 - 33.4**
Low birth weight[10]	**3.3**	**1.2 - 8.9**
Resuscitation at birth	**2.1**	**1.4 - 3.3**
Congenital anomaly	**1.8**	**1.0 - 3.3**
**Length of postnatal hospital stay ≤ 24 h**		
Maternal overweight[1]	**1.4**	**1.0 - 2.0**
Maternal age[11]	**1.5**	**1.1 - 2.1**

Parity[2]	**3.3**	**2.5 - 4.4**
Vaginal delivery	**11.9**	**3.6 - 39.5**
**Length of postnatal hospital stay ≤ 48 h**		
Parity[2]	**2.4**	**1.1 - 5.4**
Vaginal delivery	**16.9**	**7.4 - 38.7**
NICU admission	**0.09**	**0.02 - 0.3**

[1]Maternal BMI 25.00-29.99 kg/m^2;
[2]Maternal BMI ≥ 30.00kg/m^2;
[3]Caucasian ethnicity;
[4]Multiparity;
[5]Gestational age>41 weeks at delivery;
[6]Pregnancy complications including gestational hypertension, preeclampsia, and gestational diabetes;
[7]Maternal age at delivery ≥ 35 years;
[8]Nulliparity;
[9]Apgar score less than 7 at 5 min;
[10]Birth weight ≤2500 g;
[11]Maternal age < 35 years.
